# Prognostic value of neutrophil-to-lymphocyte ratio (NLR) and platelet-neutrophil (PN) index in locally advanced rectal cancer patients: a retrospective cohort study

**DOI:** 10.1097/MS9.0000000000001297

**Published:** 2024-03-18

**Authors:** Marina Morais, Telma Fonseca, Raquel Machado-Neves, Mrinalini Honavar, Ana Rita Coelho, Joanne Lopes, Emanuel Guerreiro, Silvestre Carneiro

**Affiliations:** Departments of a Surgery; b Pathologic Anatomy, Unidade Local de Saúde de Matosinhos, da Hora; Departments of c Surgery; d Pathologic Anatomy, Centro Hospitalar Universitário de São João, Porto, Portugal

**Keywords:** neoadjuvant chemoradiotherapy, neutrophil-to-lymphocyte ratio, pathological response, personalized medicine, platelet-neutrophil index, platelet-to-lymphocyte ratio, prognosis, rectal cancer

## Abstract

**Introduction:**

In locally advanced rectal cancers (LARC), tumour node metastasis (TNM) staging is far from optimal. The authors aimed to investigate the value of previously described circulating biomarkers as predictors of prognosis.

**Methods:**

Retrospective analysis of 245 LARC patients diagnosed between January 2010 and December 2022, who underwent neoadjuvant chemoradiotherapy and surgery at two centres. A Cox regression and Kaplan–Meier analysis were performed.

**Results:**

Post-treatment platelet-to-lymphocyte ratio (PLR) predicted pathological complete response. The neutrophil-to-lymphocyte ratio (NLR) in two timepoints of the treatment significantly predicted overall survival, whereas the platelet-neutrophil (PN) index significantly predicted disease-free survival. In pathological stage II, the PN index predicted patients with a higher risk of disease-free survival.

**Conclusion:**

Blood parameters might allow the definition of subgroups of risk beyond TNM for the application of different therapeutic strategies.

## Introduction

Highlights

- TNM staging is far from optimal in predicting the prognosis of locally advanced rectal cancer patients.
- Post-treatment platelet-to-lymphocyte ratio predicted pathological complete response.
- Pre-treatment and post-treatment neutrophil-to-lymphocyte ratio predicted overall survival.
- Platelet-neutrophil (PN) index predicted disease-free survival.
- Inside pathological stage II, PN index predicted a higher risk subgroup.

Colorectal cancer (CRC) is one of the most common and deadly cancers worldwide^1^. Nowadays, the standard treatment for locally advanced rectal cancer (LARC) is neoadjuvant chemoradiotherapy (nCRT) and radical surgery, including total mesorectal excision^2^. Nevertheless, due to the divergence of treatment response in LARC patients, systemic recurrence remains the main cause of treatment failure^3^. Thus, it is crucial to improve the prediction of tumour recurrence, stratification, and prognostication of these patients.

The tumour node metastasis (TNM) staging is accepted as the most powerful prognostic indicator^4^. Nevertheless, TNM staging is not completely accurate, as patients with the same-stage tumour may present different clinical outcomes^5^. Therefore, other parameters have been considered for subtyping tumours, for example, those included in the Consensus Molecular Subgroups^6^, such as microsatellite instability, hypermethylation, immune cell infiltrates, immune checkpoints, expression of certain gene pathways and metabolic dysregulation or expression of factors derived from mesenchymal-regulating angiogenesis and stromal invasion.

Inflammation is considered to be involved in tumour progression^7^, and increasing evidence has shown the correlation of blood inflammatory parameters with worse prognosis of cancers^8^. Blood circulating biomarkers have become appealing predictors of clinical outcomes because they are relatively inexpensive and reflect information on several aspects of tumour biology. Either the solitary or combined use of neutrophils, lymphocytes, monocytes, platelets, C-reactive protein (CRP), albumin, and mean corpuscular volume (MCV) have been associated with CRC survival^9–17^. However, the prognostic implication of combined ratios or scores has not been extensively evaluated in patients with LARC treated with nCRT, and there is a heterogeneity of results among multiple studies.

Therefore, this study aimed to investigate the clinical significance of several blood parameter scores, either at baseline or after nCRT, as prognostic predictors in patients with LARC.

## Methods

### Patients

This cohort study was a retrospective analysis of consecutive rectal cancer patients diagnosed in two hospitals between January 2010 and December 2022. This study was performed in line with STROCSS criteria^18^. Inclusion criteria comprised patients diagnosed pathologically with rectal adenocarcinomas, located within 12cm from the anal verge (measured by MRI), clinically staged T3-4 and/or N+ and treated with neoadjuvant chemoradiotherapy (nCRT) followed by radical surgery. Exclusion criteria were evidence of distant metastasis at diagnosis, emergent or palliative surgery, local excision or “watch and wait” approach, and evidence of inflammatory or haematological disorders. The study was approved by the Ethics Committees of both hospitals.

### Treatment and pathologic evaluation

The patients were clinically staged by magnetic resonance imaging. Patients received either one of two neoadjuvant treatment regimens, according to a multidisciplinary team decision, taking into account patient and tumour features and the point in time of treatment proposal. The standard neoadjuvant chemoradiotherapy consisted of the administration of a dose of 50.4 Gy of radiotherapy concurrent with capecitabine or 5-fluorouracil (5-FU). The most recent treatment consisted of short-course radiotherapy, with 25 Gy administered over 1 week and followed by neoadjuvant chemotherapy with combinations of 5-fluorouracil and folinic acid or capecitabine, plus oxaliplatin [total neoadjuvant therapy (TNT)].

Surgery with total mesorectal excision was scheduled for the 6th–12th weeks after completion of nCRT. Adjuvant chemotherapy was recommended selectively to patients for a period of 4–6 months.

The histopathologic examination was performed according to the classification set by the American Joint Committee on Cancer^19^. Pathological complete response (pCR) was defined as the absence of any tumour cells at the microscopic examination of the resected specimen on the final pathology^20^. All clinicopathological data were retrieved from medical records and summarized in tables.

### Follow-up

The frequency of regular outpatient consultations was once every 3 months within 2 years after curative surgery and once every 6 months in the third to fifth years. The follow-up included regular blood tests, carcinoembryonic antigen (CEA), carbohydrate antigen 19-9 (CA19-9), and chest and abdominopelvic computerized tomography. Also, a colonoscopy was recommended for patients in the first, third, and fifth years of follow-up.

### Blood markers and inflammatory scores

Pre-treatment blood samples were obtained within 2 weeks before nCRT, and post-treatment were obtained before surgery. The blood parameters recorded were haemoglobin, MCV, white blood cell count, absolute counts of neutrophils, lymphocytes and monocytes, platelets, albumin, and CEA.

Next, the most used inflammatory scores in the literature were calculated.

NLR (neutrophil-to-lymphocyte ratio) = neutrophil count / lymphocyte count.

PLR (platelet-to-lymphocyte ratio) = platelet count / lymphocyte count.

LMR (lymphocyte-to-monocyte ratio) = lymphocyte count / monocyte count.

SII (systemic immune-inflammation index) = platelet count x neutrophil count / lymphocyte count.

PN (platelet/neutrophil) index: high and low strata groups of pre-treatment platelet and neutrophil counts. The high PN index group was defined as having at least one pre-treatment platelet or neutrophil count above its respective threshold (350 ×10^9^/l for platelets and 7.5 ×10^9^/l for neutrophils).

Receiver operating characteristic (ROC) curve analysis was performed to analyze the area under the curve (AUC) and assess the scores’ optimal cut-off values.

#### Statistical analysis

The data were analyzed using SPSS 26.0 software (IBM Corporation). Clinicopathological characteristics between groups were primarily evaluated using the χ^2^ test, Student *t*-test, paired samples *t*-test, or Mann–Whitney *U* test, where appropriate. Logistic regression was performed to evaluate predictors of pCR. Overall survival (OS) was calculated as the time from diagnosis to death or last follow-up. Disease-free survival (DFS) was defined as the time from diagnosis until tumour recurrence or last follow-up. Univariable and multivariable analyses (Cox regression model) were used to assess risk factors of the outcomes. Survival outcomes were analyzed using the Kaplan–Meier method, and comparisons were assessed using the log-rank test. A two-sided *P* less than 0.05 was considered statistically significant.

## Results

### Patient characteristics

A total of 245 patients were included in our analysis, 200 (81.6%) from one centre and 45 (18.4%) from another. There were few significant differences between the clinicopathologic features of patients from both hospitals, except for the Charlson score, clinical T stage, and interval of time between neoadjuvant treatment and surgery. The clinicopathological features of all patients are shown in Table 1. There were 151 (61.6%) males and 94 (38.4%) females, with 61 ± 12 years on average. Most patients had clinical stage III disease (80.4%), and the majority received nCRT (89.4%), with only 8.2% receiving a TNT regimen. Most patients had a stoma performed (91.8%). Of the only 19 patients submitted to anterior rectal resection without a stoma, only 4 presented a leak. The pCR rate was 14.7%. There were 42 missing cases for circumferential rectal margin, 22 for distal rectal margin, and mesorectal complete excision was only available in 48.9% of the patients, and therefore, we decided not to include this variable in the Cox regression analysis.

**Table 1:** Clinicopathological features

Parameters	Values
Patient characteristics	
Sex	
Male	151 (61.6)
Female	94 (38.4)
Age, years	61 ± 12
Charlson score	
0–1	116 (47.3)
≥ 2	129 (52.7)
BMI	
<25	100 (40.8)
≥ 25	126 (51.4)
Tumour features	
Dimensions (longitudinal)	
< 5 cm	129 (52.7)
≥ 5 cm	99 (40.4)
Clinical stage	
II	48 (19.6)
III	196 (80.4)
Treatment	
Neoadjuvant chemotherapy	
Capecitabine / 5-FU	219 (89.4)
TNT	20 (8.2)
Interval neoadjuvant treatment to surgery, weeks	10 (3–51)
Surgery	
Anterior resection	167 (68.2)
with stoma	148 (88.6)
Abdomino-perineal amputation	78 (31.4)
Approach	
Open	112 (45.7)
Laparoscopic	133 (54.3)
Total postoperative complications	58 (23.7)
Intra-abdominal complications (leaks, abscesses)	25 (10.2)
Adjuvant chemotherapy	142 (58.0%)
Histopathological features	
Histological stage	
II	179 (73.1)
III	66 (26.9)
Grade	
1	20 (8.2)
2	157 (64.1)
3	2 (0.8)
Vascular invasion	72 (29.4)
Perineural invasion	59 (24.1)
Margins	
R 0	214 (87.3)
R 1–2	30 (12.2)
CRM, cm	1 (0–10)
DRM, cm	3,5 (0–33.5)
Mesorectal complete excision	59 (24.1)
Pathologic regression grade (Ryan *et al*.^21^)	
1	107 (43.7)
2-3	125 (51.1)
pCR	26 (14.7)
Baseline and post-nCRT blood parameters	
CEA (ng/dl)	4.17 (0.50–358)
Haemoglobin (g/dl)	13.5 (7.3–17.3) → 12.7 (7.9–16.2)
MCV (fl)	89.3 (64–110) → 91.9 (43–106)
Leucocytes (×10^9^/l)	7.88 (3.2–18.5) → 5.52 (1,95–26)
Neutrophils (×10^9^/l)	4.92 (1.80–11.9) → 3.9 (0.5–25)
Lymphocytes (×10^9^9/l)	2.00 (0.40–5.80) → 0.83 (0.3–9)
Monocytes (×10^9^/l)	0.60 (0.21–1-44) → 0.5 (0.18–5.8)
Platelets (×10^9^/l)	243 (66–719) → 212 (40–632)
Outcomes	
Follow-up, years	4 (0×11)
3-year OS	81.7%
3-year DFS	69.5%
5-year OS	69.9%
5-year DFS	65.6%
Loco-regional recurrence	14 (5.7)
Systemic recurrence	63 (25.7)

Values are presented as *n* (%), mean ± s.d. or median (range).

CEA, carcinoembryonic antigen; CRM, circumferential resection margin; DFS, disease-free survival; DRM, distal resection margin; MCV, mean corpuscular volume; nCRT, neoadjuvant chemoradiotherapy; OS, overall survival; pCR, complete pathological response; TNT, total neoadjuvant therapy.

The medians (range) of blood markers values are also presented in Table 1. Post-treatment CEA was not available in most patients, so we decided not to include it. Forty-two (18.4%) patients and 32 (13.1%) had a high PN index pre and post-treatment, respectively. The median pre-treatment NLR, PLR, LMR and SII levels were 2.47 (0.68–10.79), 128.0 (16.5–801.22), 3.33 (0.40–14.14) and 612 (102.5–7090.8), respectively. The median post-treatment NLR, PLR, LMR and SII levels were 4.50 (0.39–56.67), 257.1 (27.4–969), 1.73 (0.34–9.42) and 926 (98.2–20456), respectively. A paired samples *t*-test was used to evaluate the difference between means of pre-treatment and post-treatment blood markers and scores, and in all the difference in means was statistically significant.

### Predictive and prognostic value of circulating blood parameters

**Figure 1:**
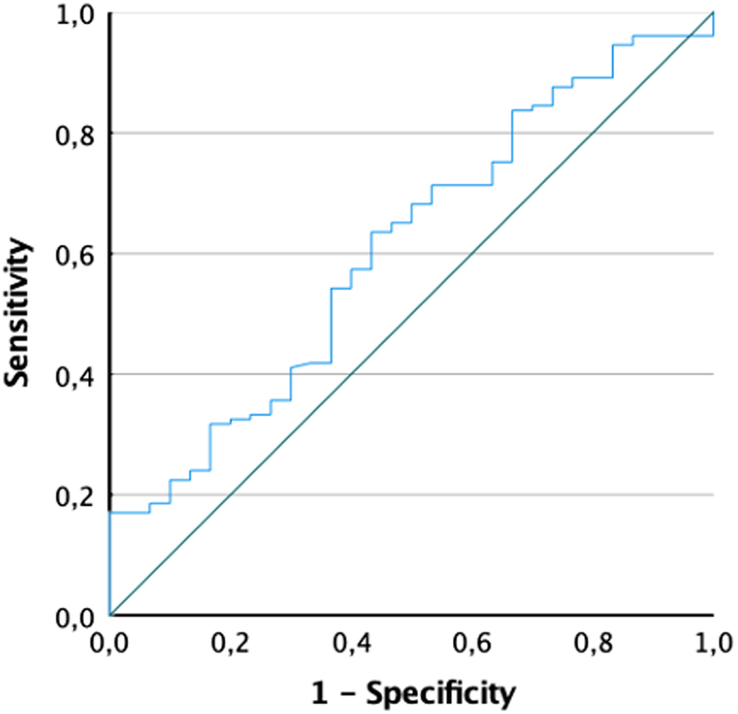
Receiver operating characteristic curve of value of post-neoadjuvant chemoradiotherapy platelet-to-lymphocyte ratio for prediction of pathological complete response.

A logistic regression was performed to evaluate predictors of pCR, shown in Table 2. On multivariable analysis, male sex [odds ratio (OR) 4.731, *P*<0.001] and higher tumour length (OR 1.402; *P*=0.010) were predictors of incomplete response. Furthermore, post-nCRT PLR also appeared as an independent factor of non-pCR [OR 1.005 (10.001–1.009, *P*=0.008)]. We performed a ROC curve for its value on pCR and obtained an AUC of 0.616, *P*=0.033, with a value of PLR of 230 corresponding to a sensitivity of 60% and specificity of 57.6% (Fig. 1).

**Table 2:** Logistic regression analysis of significant predictors of pCR to nCRT in LARC

	pCR (odds ratio and 95% CI; *p* value)			
Variable	Univariable	*P*	Multivariable	*P*
Age	0.995 (0.965–1.025)	0.723		
Sex (male)	**3.439** (**1.644**–**7.192)**	**0.001**	**4.731** (**1.986**–**11.271)**	**>0.001**
Tumour length	**1.357** (**1.104**–**1.668)**	**0.004**	**1.402** (**1.085**–**1.811)**	**0.010**
cT (>2)	**0.279** (**0.088**–**0.887)**	**0.031**	—	—
cN (+)	3.037 (0.890–10.360)	0.076	2.860 (0.741–11.045)	0,127
Interval nCRT-surg	1.015 (0.940–1.095)	0.712	—	—
Post-nCRT PLR	**1.004** (**1.001**–**1.007)**	**0.021**	**1.005** (**1.001**–**1.009)**	**0.008**

Bold indicate statistically significant results.

CEA, carcinoembryonic antigen; LARC, locally advanced rectal cancer; LMR, lymphocyte-to-monocyte ratio; MCV, mean corpuscular volume; nCRT, neoadjuvant chemoradiotherapy; pCR, pathological complete response; PLR, platelet-lymphocyte ratio.

Significant in univariable but not evidenced in the final model: post-nCRT NLR, post-nCRT SII.

Non-significant in univariable or multivariable analysis: tumour location, CRT regimen, blood parameters and scores not included in the table: haemoglobin, MCV, CEA, LMR, pre-nCRT NLR, PLR and SII.

The median follow-up was 4 years (ranging from 0 to 11). The 3-year OS was 81.7% and DFS was 69.5%. These lowered to 69.9% and 65.6% at the 5-year mark, respectively. Fourteen patients (5.7%) presented loco-regional recurrence and 63 patients (25.7%) recurred with distant metastasis. According to pathological response, 5-year OS was 81.0% for pCR compared to 68.3% for non-pCR patients (*P*=0.080), and 5-year DFS was 91.7% and 61.6%, respectively (*P*=0.004).

Table 3 shows the analysis for predictors of prognosis. Multivariable analysis revealed that greater longitudinal dimensions of the tumour, pathological stage III, vascular invasion, absence of adjuvant chemotherapy higher, and pre-nCRT CEA were significantly associated with mortality. Furthermore, higher pre-nCRT and post-nCRT NLR led to a worse prognosis.

**Table 3:** Univariable and multivariable analysis (Cox Regression) of predictors of overall survival and disease-free survival

	OS (HR and 95% CI; *p* value)				DFS (HR and 95% CI; *p*)			
Variable	Univariable	*P*	Multivariable	*P*	Univariable	*P*	Multivariable	*P*
Sex: Male	0.922 (0.575–1.479)	0.736			0.753 (0.467–1.216)	0.247		
Age	0.999 (0.979–1.020)	0.938			0.983 (0.964–1.002)	0.074		
Charlson	1.035 (0.905–1.184)	0.613	—	—	0.949 (0.824–1.093)	0.465		
BMI	**0.933** (**0.880**–**0.988)**	**0.018**	—	—	0.980 (0.931–1.033)	0.454		
Clinical stage III	0.882 (0.521–1.491)	0.638	—	—	0.713 (0.423–1.200)	0.202	—	—
Dimensions	1.004 (0.898–1.123)	0.944	**0.842** (**0.716**–**0.991)**	**0.039**	0.969 (0.865–1.086)	0.592	**0.819** (**0.718**–**0.935)**	**0.003**
Interval treatment surgery (weeks)	1.022 (0.982–1.065)	0.283	—	—	0.991 (0.944–1.040)	0.711	—	—
Surgery APR	1.435 (0.906–2.271)	0.124	—	—	**1.956** (**1.246**–**3.072)**	**0.004**	**2.518** (**1.406**–**4.511)**	**0.002**
Surgical morbidity	1.277 (0.779–2.092)	0.333			1.252 (0.754–2.079)	0.384		
Pathological stage III	**3.172** (**2.007**–**5.012)**	**<0.001**	**4.763** (**2.092**–**10.846)**	**<0.008**	**3.114** (**1.980**–**4.897)**	**<0.001**	**2.584** (**1.440**–**4.636)**	**0.001**
Vascular invasion	**2.708** (**1.715**–**4.276)**	**<0.001**	**2.419** (**1.162**–**5.034)**	**0.018**	**3.615** (**2.272**–**5.753)**	**<0,001**	—	—
Perineural invasion	**3.270** (**2.055**–**5.202)**	**<0.001**	—	—	**3.582** (**2.253**–**5.692)**	**<0.001**	**2.400** (**1.299**–**4.437)**	**0.005**
R≥1	**4.066** (**2.387**–**6.927)**	**<0.001**	—	—	**2.357** (**1.334**–**4.165)**	**0.003**	—	—
Non-pCR	2.201 (0.887–5.457)	0.089	—	—	**4.559** (**1.437**–**14.466)**	**0.010**	—	—
CRM	**0.698** (**0.556**–**0.909)**	**0.008**	—	—	0.878 (0.707–1.089)	0.237	**1.216** (**1.035**–**1.428)**	**0.017**
DRM	1.006 (0.956–1.059)	0.806	—	—	1.033 (0.998–1.070)	0.068	—	—
No adjuvant CT	1.338 (0.836–3.267)	0.238	**3.239** (**1.530**–**6.855)**	**0.002**	1.128 (0.700–1.816)	0.621	—	—
Pre-nCRT CEA	**1.006** (**1.002**–**1.010)**	**0.003**	**1.010** (**1.004**–**1.016)**	**<0.001**	1.008 (0.991–1.024)	0.367		
Pre-nCRT NLR	**1.178** (**1.019**–**1.361)**	**0.027**	**1.298** (**1.032**–**1.632)**	**0.026**				
Post-nCRT NLR	**1.036** (**1.003**–**1.070)**	**0.033**	**1.058** (**1.008**–**1.111)**	**0.022**				
Post-nCRT PN index					**2.240** (**1.289**–**3.894)**	**0.004**	**2.967** (**1.470**–**5.991)**	**0.002**

Bold indicate statistically significant results.

All remaining clinical data, such as nCRT regimen, blood parameters and scores studied in this work were not independent predictive factors of prognosis in this analysis. We did not include the parameter mesorectal complete excision, as about half of the patients had missing cases of these parameters.

APR, abdominal-perineal resection; CEA, carcinoembryonic antigen; CRM; circumferential resection margin; CT, chemotherapy; DFS, disease-free survival; DRM, distal resection margin; HR, hazard ratio; nCRT, neoadjuvant chemoradiotherapy; NLR, neutrophil-to-lymphocyte ratio; OS, overall survival; pCR, pathological complete response; PN index, platelet-neutrophil index.

As for DFS, greater tumour dimensions, APR patients, pathological stage III, perineural invasion, and higher CRM were associated with more recurrences. Also, higher PN index was an independent factor of recurrence, mainly for loco-regional recurrence (hazard ratio 6.909, *P*<0.018), as no statistical significance was achieved for distance metastasis. The analysis of other circulating parameters and prevalent scores in literature showed that they were not independent predictive factors of prognosis in this analysis.

**Figure 2:**
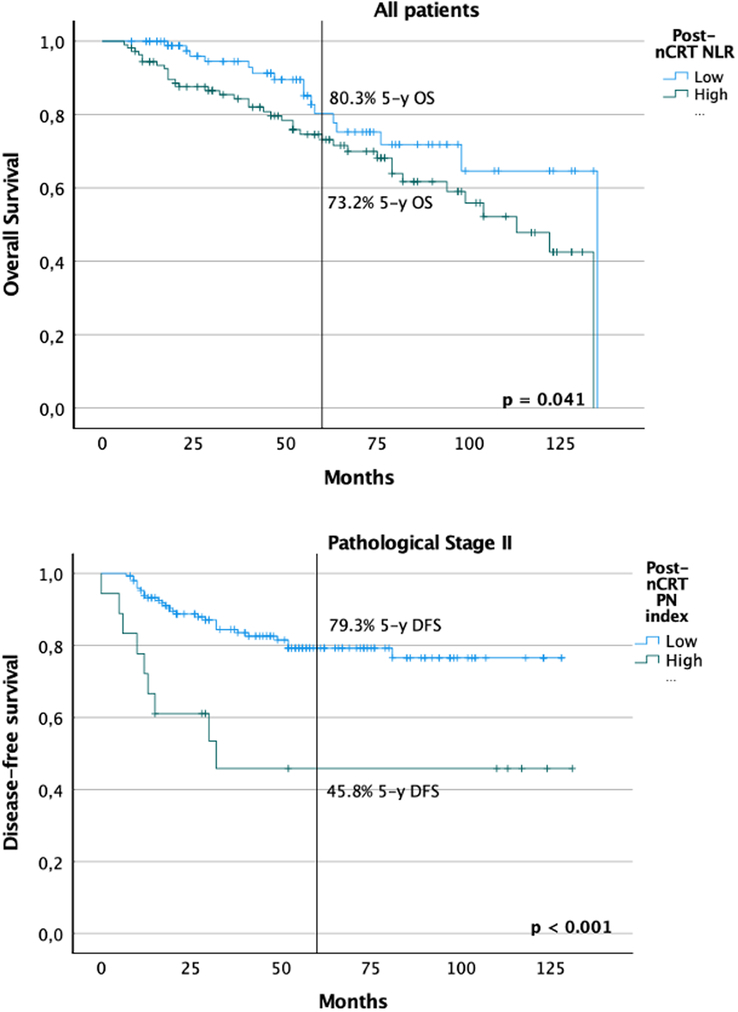
Kaplan–Meier curves for overall survival and disease-free survival stratified by the scores retrieved from multivariable analysis (after R1–2 patients excluded). nCRT, neoadjuvant chemoradiotherapy; NLR, neutrophil-to-lymphocyte ratio; PN, platelet-neutrophil.

Finally, a Kaplan–Meier survival analysis was performed to study the impact of the factors found to be significant predictors of survival in the previous analysis of subtyping LARC patients. These were studied using ROC curve analysis to find the most accurate cut-off value for the prediction of these outcomes. A high pre-nCRT NLR value was considered above 2.5, and a high post-nCRT NLR value above 4 for OS. Patients with affected resection margins (30 patients, 12.2%) were excluded from this analysis. Although results for pre-nCRT were non-significant, we found subgroups of different prognoses for post-nCRT NLR (*P*=0.041). Moreover, the post-nCRT PN index was able to differentiate two subgroups with different disease-free survival (*P*=0.014), even inside pathological stage II (*P*<0.001) (Fig. 2).

## Discussion

In this study, we found that pre- and post-nCRT NLR were independent factors of OS, and that post-treatment PN index was an independent factor of DFS in LARC patients. This was one of the few studies to investigate and evidence the value of several scores at different timepoints in the prognosis of LARC patients. However, other parameters well described in the literature, such as PLR, LMR and SII could not achieve significance in this study. Also, pCR was previously correlated with a better prognosis^22^. In our analysis, although not an independent factor, it has shown some impact on survival. A lower post-nCRT PLR was independently associated with pCR, although with moderate accuracy. This association has been observed previously: one study^23^ has shown that high pre-nCRT platelet levels, changes in platelet levels, and post-nCRT PLR were associated with worse pathological response, whereas another report^24^ showed that high intra-nCRT and high PLR change rate were predictive of pCR.

The host systemic response plays a significant role in cancer development and progression^7^. Here, we have found an association between worse OS and NLR, an integrated index based on peripheral neutrophils and lymphocytes. The value of NLR in the prognosis of rectal cancer has been extensively described previously^25^, although these results should be read with caution since a large-scale study of 1237 patients could not confirm the prognostic or predictive value of NLR or PLR^26^. Nevertheless, in our study, this score performed better than other scores described in the literature, such as PLR, LMR and SII^27^. Regarding disease-free survival, the post-nCRT PN index, a composite index of platelets and neutrophils, proved to have an impact on our study. These scores allowed the definition of subgroups of risk as shown by the Kaplan–Meier method. Also, inside pathological stage II, in which treatment is still controversial, we could define further subgroups of disease-free survival using the PN index. Patients in pathological stage II were proposed for adjuvant computed tomography (CT), mainly taking into account the pT stage and co-morbidities (Charlson score). The definition of subgroups of prognosis in these patients could help individualize treatment and potentially alter their strategy.

Neutrophils secrete cytokines and chemokines that play an important role in cancer progression^28^, and neutrophilia has been shown to predict inferior outcomes in rectal cancer^29^. Lymphopenia is also commonly present in these tumours and might help tumour cells escape immune surveillance and prevent damage by cytotoxic T cells^30^. Thus, a high NLR level reflects alterations in the cancer microenvironment that favour cancer initiation and progression. High NLR has been previously associated with a worse response to nCRT^31,32^, which could help explain why it might be associated with a worse prognosis. Platelets are thought to be involved in tumour progression by regulating neo-angiogenesis and by aiding circulating tumour cells to escape immune surveillance^33,34^. Several meta-analyses have confirmed that a high NLR and high PLR are significant prognostic factors for poor outcomes in LARC^35,36^. PN index, an index of neutrophils and platelets present in the previous scores mentioned, could also have an impact on the prediction of DFS, although this association has not been reported before and deserves further consideration.

To improve DFS and OS and optimize the treatment of patients with LARC, new approaches to treatment and staging are emerging. Several trials studied TNT, in which systemic induction chemotherapy precedes nCRT and surgical resection. In the recent RAPIDO trial^37^, the experimental TNT group increased the rate of pCR and decreased the rate of distant metastases compared to the conventional approach. On the other hand, some studies have shown that an “immunoscore”^38^, calculated with counts of CD8+ or FoxP3+ regulatory T cells, outperformed the traditional TNM staging in predicting outcomes in CCR^39^ and a plethora of immune checkpoint inhibitors and other immunotherapies are emerging^40^. Therefore, it seems relevant to assess the impact of inflammatory markers in the subtyping of disease to select the best strategy for each patient.

There are some limitations to our research. This study was a retrospective analysis with a relatively small sample that included two different neoadjuvant chemoradiation regimens. However, two hospital populations are represented, and the results of blood parameters collected in 2 timepoints are presented. In addition, we report that patients with inflammatory or haematological conditions were excluded, but patients with other chronic diseases (such as cardiomyopathy, heart diseases, kidney disease and peripheral arterial disease) were included in order to maintain a substantial sample, although we understand these pathologies could affect the inflammatory condition of the patient and be a confounding variable. Still, the Charlson score, composed by these co-morbidities, was analyzed in this study and did not have an independent impact on the outcomes. Also, CRP, albumin and post-nCRT CEA, which has shown prognostic implications in literature, were not routinely examined in both centres, and there is a lack of clearly defined cut-off value for inflammatory markers in the literature. Nevertheless, our study adds to the understanding of the prognostic implication of circulating blood parameters in patients with LARC after nCRT due its long follow-up and evaluation of several baseline parameters. Patients with high NLR in two timepoints and post-treatment PN index might correspond to higher risk groups of stratification and could be proposed for a closer follow-up and more aggressive treatment than those with lower values.

## Conclusion

Measurement of circulating parameters at different timepoints across neoadjuvant treatment might enable tailored strategies and prognostication and consequently improve survival outcomes in LARC patients. However, these results require further validation in larger prospective populations.

## Ethical approval

The study was approved by the Ethics Committee of both hospitals.

## Consent

This is a retrospective study that used data from patients for analysis, therefore consent from patients was not applicable.

## Source of funding

This research received no specific grant from any funding.

## Author contribution

M.M. and T.F. retrieved all data. R.M.N., M.H., A.R.C. and J.L. reviewed the histopathological results. M.M. analyzed the data and was the major contributor in preparing the manuscript. All authors reviewed and approved the final manuscript.

## Conflicts of interest disclosure

The authors declares that they have no conflicts of interest.

## Research registration unique identifying number (UIN)

Clinicaltrials.gov NCT05893667 https://clinicaltrials.gov/show/NCT05893667.

## Guarantor

Marina Morais, Silvestre Carneiro.

## Data availability statement

The datasets generated for this study are available upon reasonable request.

## Provenance and peer review

Not commissioned, externally peer-reviewed.
